# Knowledge, Attitude, and Practices Related to the SARS-CoV-2 Pandemic among Women Seeking Contraceptive Methods

**DOI:** 10.1055/s-0041-1741448

**Published:** 2022-05-27

**Authors:** Elaine Aparecida Lopes Garcia, Jessica Mayra Ferreira, Nelio Veiga-Junior, Luis Bahamondes, Ilza Monteiro

**Affiliations:** 1 Department of Obstetrics and Gynecology, Universidade Estadual de Campinas, Campinas, SP, Brazil

**Keywords:** COVID-19, knowledge, attitudes, practices, copper IUD, levonorgestrel intrauterine system, socio demographic characteristics, COVID-19, conhecimento, atitudes, práticas, DIU de cobre, sistema intrauterino de levonorgestrel, características sociodemográficas

## Abstract

**Objective** To determine knowledge, attitude, and preventive (KAP) practices towards the SARS-CoV-2 (COVID-19) pandemic among women in reproductive age seeking to use copper or hormonal intrauterine devices (IUD/LNG-IUS).

**Methods** We conducted a cross-sectional study in which we applied a questionnaire on 400 women about KAP practices on COVID-19 at the University of Campinas, Campinas, SP, Brazil, from May to August 2020.

**Results** The mean (±SD) age of the women was 30.8 ± 7.9 years, and 72.8% of them reported being pregnant at least once. Most women (95%) had heard or read about COVID-19, and their main sources of information were television (91%) and government websites (53%). However, 53% of the women had doubts about the veracity of the information accessed.

**Conclusion** Women without a partner and with > 12 years of schooling had more information about COVID-19 and on its impact on new pregnancy, and those from high socioeconomic status had a higher chance of maintaining physical distance. Safety, effectiveness, comfort, and absence of hormone in the contraceptive method (in the case of TCu380A IUD) were the main reasons for the participants to seek the service during the pandemic, and the possibility to stop menstrual bleeding was the main reason to choose the LNG-IUS.

## Resumo

**Objetivo** Determinar o conhecimento, atitude e práticas preventivas (CAP) em relação à pandemia de SARS-CoV-2 (Covid-19) entre mulheres em idade reprodutiva que buscam usar dispositivo intrauterino com cobre (DIU TCu 380) ou sistema intrauterino liberador de levonorgestrel (SIU-LNG).

**Métodos** Foi realizado um estudo transversal e um questionário foi aplicado a 400 mulheres para conhecer o CAP sobre o COVID-19 na Universidade Estadual de Campinas, Campinas, SP, Brasil, no período de maio a agosto de 2020.

**Resultados** A média (±DP) de idade das mulheres foi de 30,8 ± 7,9 anos, e 72,8% delas relataram ter engravidado pelo menos uma vez. A maioria das mulheres (95%) tinha ouvido ou lido sobre a a Covid-19, e suas principais fontes de informação foram a televisão (91%) e sites do governo (53%). Porém, 53% das mulheres tinham dúvidas a respeito da veracidade das informações acessadas.

**Conclusão** Mulheres sem companheiro e com mais de 12 anos de escolaridade tiveram mais informações sobre a COVID-19 e sobre o seu impacto em uma nova gravidez, e aquelas de nível socioeconômico alto tiveram maior chance de manter distância física. Segurança, eficácia, conforto e ausência de hormônio no método anticoncepcional (no caso do DIU TCu380A) foram os principais motivos para as participantes procurarem o serviço durante a pandemia, e a possibilidade de controlar o sangramento menstrual abundante foi o principal motivo para a escolha do SIU-LNG.

## Introduction

The SARS-CoV-2, which provoked the COVID-19 pandemic by a new coronavirus, is a threatening and highly contagious infection. It was first detected in December 2019 in the province of Wuhan, China. 1 2 3 On March 11, 2020, the World Health Organization (WHO) declared COVID-19 as the largest and fastest-growing pandemic of the century. 3 Several actions have been taken to try to contain the spread of the virus, such as physical distancing, isolation, lockdown, and quarantine. Regarding healthcare services, many policy makers decided to maintain only essential services due to the lack of personnel, because they were allocated to provide health service related to COVID-19. 4

Sexual and reproductive health (SRH) services, including contraceptive counseling and provision, and abortion services were considered non-essential by many policy makers, and several clinics that provide SRH services were temporarily closed. 1 2 5 For this reason and due to the interruption of chain supplies, the United Nations Population Fund reported that a shortage of contraceptive methods is expected in many settings as well as an increase in unplanned pregnancies and abortions, which could be unsafe in many settings in the near future. 6 7 This scenario shows that women and men are even more in need of contraceptive methods during the pandemic. 8 9

Although many clinics are closed, several measures can be taken to continue the provision of contraceptives, including long-acting reversible contraceptive (LARC) methods. It is possible to implement remote consultation and counseling in particular settings to avoid unnecessary visits to the clinic, provide electronic prescriptions and more time between consultations when face-to-face appointments are needed. 1 9 10 11 In the family planning clinic based in the University of Campinas, Brazil, we continued to assist women during the pandemic by offering counseling and provision of contraceptive methods (mainly LARC methods) with proper authorization of local authorities. We implemented all possible safety measures to reduce the risk of COVID-19 contamination, such as telemedicine for women, when possible; reduction of daily appointments; more time between consultations; brief procedure visits; provision of open waiting spaces outside the facility, and use of appropriate personal protective equipment. 11 12

We considered that there is a high probability that more women are trying to avoid pregnancy during the pandemic, and they have sought the family planning services looking for safe and effective contraceptive methods like LARCs. For this reason, our primary objective was to assess the knowledge, attitude, and preventive (KAP) practices towards COVID-19 among women in reproductive age. The secondary aim was to assess the reasons why these women came to the clinic seeking to use the copper-intrauterine device (Cu-IUD) or the levonorgestrel 52 mg intrauterine system (LNG-IUS).

## Methods

### Study Design and Participants

This was a cross-sectional study carried out at the department of obstetrics and gynecology of University of Campinas Medical School (UNICAMP), Campinas, SP, Brazil. The protocol was approved by the Ethical Committee of the university, and all women signed an informed consent before they were admitted to the study. Women between 18 and 49 years of age who visited the family planning clinic from May to August 2020 were invited to participate in the study and were included if they were willing to use the TCu380A IUD (Optima, Injeflex, São Paulo, Brazil) or the LNG-IUS (Mirena, Bayer Oy, Turku, Finland). Women who wanted to use the LNG-IUS only as a therapy for abnormal uterine bleeding were excluded. The participants filled out a 37-item questionnaire specially developed for the purpose of our study, which contained sociodemographic information and questions regarding their KAP practices towards COVID-19. The questionnaire was based on the WHO training material for detection, prevention, and control of the disease. 13 14 It was comprised of multiple choice and open-ended questions, including 16 questions about knowledge (6 of them regarding contraceptives), 6 questions about attitude, and 6 questions about preventive practice.

### Analysis of the Data

We estimated the sample size assuming that 60% of the participants would be well informed about the COVID-19 pandemic and came to a sample of 327 women in the study. The participants' sociodemographic characteristics were presented as descriptive with mean (±standard deviation [SD]), or as percentage distribution for categorical variables using the χ ^2^ or Fisher exact test for comparison. The univariate analysis followed by linear regression analysis (with stepwise analysis) was used to determine the relation between demographic variables and the correct answers provided by the women. Non-standardized regression coefficients (95%) were used to assess the correlation between the answers towards COVID-19. We used the SAS 9.4 software (SAS Institute, Cary, NC, USA). The level of significance was set at *p* < 0.05.

## Results

Among the 410 women invited to participate in the study, 400 returned the questionnaire and were included in the analysis. The participants' mean (±SD) age was 30.8 ± 7.9 years. A rate of 56.1% of the women had a history of 1 to 2 pregnancies, 75.9% had more than 12 years of schooling, and 55.1% were living with a partner. The participants were allowed to check more than one contraceptive method in use in the questionnaire at the time of the consultation. Excluding 255 women (63.7%) that reported the use of condoms, a LARC method was used by 109 women (27.2%) followed by 100 (25.0%) users of combined oral contraceptives ( Table 1 ). All the users of LARCs who came to the clinic looking for an intrauterine device/system (IUD/IUS) were women who planned to replace the IUD after the approved labeled time of use (data not shown).

**Table 1:** Sociodemographic characteristics of respondents, contraceptive methods in use at the time of interview and its length of use

Variables	n (%)
Age (years) (n = 393)	
< 20	22 (5.6)
20 a 29	164 (41.7)
30 a 40	145 (36.9)
≥ 40	62 (15.8)
Ever pregnant (n = 312)	
0	85 (27.2)
1-2	175 (56.1)
> 3	52 (16.7)
Cohabitation status (n = 390)	
With partner	215 (55.1)
Without partner	175 (44.9)
Ethnicity (n = 393)	
White	206 (52.4)
Black	141 (35.9)
Biracial	40 (10.2)
Asian	6 (1.5)
Schooling (years) (n = 378)	
< 1	3 (0.8)
1–4	2 (0.5)
5–9	12 (3.2)
10–12	74 (19.6)
≥ 13	287 (75.9)
Number of children (n = 304)	
0	92 (30.3)
1–2	186 (61.2)
≥ 3	26 (8.5)
Familiar income (US Dollar amount) (n = 390)	
R$ 403 (US$ 72)	14 (3.6)
R$ 618 (US$ 110)	18 (4.6)
R$ 933–1,391 (US$ 166–250)	161 (41.3)
R$ 2,327–4,558 (US$ 416–815)	156 (40.0)
R$ 8,099–14,366 (US$ 1,430–2,570)	41 (10.5)
Occupation (n = 376)	
Unemployed	100 (26.6)
Formal/informal job	236 (62.8)
Housewife	40 (10.6)
Number of family members at home (n = 375)	
1–3	218 (58.1)
4–6	151 (40.3)
≥ 7	6 (1.6)
Contraceptive in use (more than one answer allowed) (n = 400)	
Condom	255 (63.7)
Combined oral contraceptives	100 (25.0)
Injectables	33 (8.2)
Intrauterine devices/implants	109 (27.2)
Permanent contraception	7 (1.7)
No method	24 (6.0)
Fertility awareness methods	24 (6.0)

When asked about the COVID-19 pandemic, 95% of the women had heard or read information about it and 57% of them found it difficult to decide if the information about the disease was reliable ( Table 2 ). Television was the main source of information among the participants (91%), followed by government websites (53%), radio (45%), and social media (20%). In addition, the participants reported fever (89.8%), difficulty breathing (85.2%), cough (76.2%), loss of sense of smell (69.5%), headache (65.2%), and sore throat (50.0%) as the most important signs and symptoms of the COVID-19 infection. When asked how severe the virus could be to a pregnant woman and the fetus, 46% of women believed it was as serious as the Zika virus, while 22.1% and 34.0% reported not having enough information about the consequences of COVID-19 to pregnant women and the fetus, respectively.

**Table 2:** Some knowledge of the participants about Covid-19

	n (%)
Have you ever heard about covid-19? (n = 400)	
Yes	379 (94.7)
No	21 (5.3)
Have you read any message about covid-19? (n = 400)	
Yes	380 (95.0)
No	20 (5.0)
It is difficult to decide if the information about the coronavirus is true, false or rumors (n = 351)	
Disagree	65 (18.5)
Neither agree nor disagree	86 (24.5)
Agree	200 (57.0)
A person who is not sick or who does not have symptoms cannot spread the coronavirus (n = 363)	
Disagree	242 (66.7)
Neither agree nor disagree	35 (9.6)
Agree	86 (23.7)

Regarding the women's attitude towards the pandemic, 62.2% were concerned and 36.4% were afraid or very afraid of it. More than ⅓ (39.2%) of the participants considered they were capable of protecting themselves from contamination; however, the reduction of their usual income (24.9%) and difficulty of being socially isolated (62.2%) were the main reasons that interfered with the participants' actions to protect themselves. A rate of 50.0% of participants agreed that the changes in habits are important to avoid further dissemination of the COVID-19, 108 (29.3%) of the women were indifferent about it, and 12.7% disagreed with this statement. More than ⅔ (77.6%) of the participants took action to avoid an unplanned pregnancy during the pandemic. Regarding the preventative practices, 99.2% of the participants took some action to protect themselves against the contamination and spread of the virus. The most common practices to avoid COVID-19 contamination included wearing a mask when leaving home (88.7%), using alcohol gel to clean their hands (86.7%), avoiding leaving home (81%), and keeping a distance of 1 to 2 meters from other individuals (70.5%) ( Table 3 ).

**Table 3:** Some practices to avoid Covid-19 and to avoid pregnancy by the respondent women (more than one option was allowed)

Practice to avoid coronavirus (n = 400)	n (%)
Wash hand for 20 seconds	268 (67.0)
Use alcohol gel for hand hygiene	347 (86.7)
Wear a mask outside home	355 (88.7))
Cleaning the house with disinfectants	254 (63.5)
Maintain 1–2 meters of social distance	282 (70.5)
Avoid touch face	218 (54.5)
Avoid leaving home	324 (81.0)
**Practice to avoid pregnancy (n = 334)**	**n (%)**
Use condom	89 (26.6)
Avoid sexual intercourse	65 (19.5)
Use fertility awareness method	15 (4.5)
Looking for a new contraceptive	38 (11.4)
No new attitude	127 (38.0)

The main reasons that motivated women to come to the clinic requesting the use of an IUD/IUS were to avoid pregnancy (40.7%), for personal reasons (19%), contraception and abnormal uterine bleeding (17.5%), abdominal lower pain (15.6%), and indication for medical treatment in addition to contraception (9.6%). The TCu380A IUD and the LNG-IUS were chosen by 33.9% and 66.1% of the participants, respectively. The main reason for their choice were safety (56.6%), the lack of daily attention required (38.1%), decrease or interruption of uterine bleeding in the case of the LNG-IUS (34.4%), and the absence of hormones in the case of the TCu380A IUD (21.7%). Although 72.5% of the women reported being concerned about becoming pregnant during the pandemic, the majority (82%) responded that the pandemic did not influence their choice for an IUD. After the logistic regression analysis, we observed that the women who reported having heard more information about COVID-19 and its impact on pregnancy were those without a partner and with more than 12 years of schooling (2.8 and 5.5 times higher than those without a partner and with < 12 years of schooling, respectively). In addition, women from low socioeconomic status were 16 times more likely to be influenced by the pandemic in their choice for an IUD when compared with women in higher socioeconomic levels. Women from high socioeconomic status and those with a partner had, respectively, 14.6 and 1.8 more chance to maintain physical distancing than the participants in the lower economic classes. Women older than 40 years of age had 15.4 times more chances to avoid leaving home than those aged under 20 years old.

## Discussion

We found that most of the women who came to the clinic during the COVID-19 pandemic avoided leaving home and considered that the disease was a serious problem for the population. However, less than 30% of our sample had a job outside home making it possible for many women to stay home.

The COVID-19 pandemic raised a controversial question on whether women should consider postponing pregnancy due to the potential risk related to vertical transmission. 15 16 Since it became evident that there could be perinatal transmission with the HIV 17 epidemic, and the discussion about the subject resurfaced with the arrival of H1N1, in 2009, and the Zika virus, in 2016 and 2017, the possibility of vertical transmission of COVID-19 during pregnancy is an important consideration. 3 Although available data on risks related to COVID-19, specifically on vertical transmission of the virus, are limited, a recent study found that pregnant women with COVID-19 are 1.5 times more likely to be admitted to an intensive care unit (ICU) and 1.7 times more at risk of requiring mechanical ventilation compared to non-pregnant women of childbearing age with COVID-19. However, pregnant women were not at increased risk of death. 15 16

However, it was reported that the risk of death by COVID-19 was higher in pregnant women in Brazil than in other countries, especially among women with comorbidities like preeclampsia and obesity, which are very common conditions in our population. 17 Although vertical transmission of COVID-19 is apparently rare, the evidence suggests that premature birth and admission to a neonatal ICU are common among babies born to women infected with COVID-19. 15 16

The women who came to our service during the pandemic reported more years of schooling than the common population attended at the clinic, which could explain why they were well-informed about the most common symptoms of COVID-19. The knowledge about the virus was obtained mainly through television, probably because families spent more time at home during the quarantine and had more opportunities to watch television. 18 19 In addition, the fact that many healthcare facilities were closed or only dedicated to attend people with COVID-19 constituted a barrier for many women from the underprivileged portion of the society from receiving information from healthcare providers. 7 20

Family income was associated with a polarization of our sample in the responses. Women who belonged to classes A and B questioned the information received about COVID-19, while a great rate of women in classes C, D, and E reported no opinion on the reliability of the information they received. We found that the possibility to take action to face the pandemic was directly proportional to the high income and years of schooling of the participant. 21 In Brazil, the government provided conflicting information about the pandemic. The Ministry of Health and many governors encouraged physical distance, the use of masks and lockdown. Conversely, the Brazilian president encouraged people to not practice physical distancing and to not stay home. 22 These conflicting messages from the highest level of administration created a confusing scenario for many people in the middle of the pandemic. For this reason, we believe that women with fewer years of schooling and with lower income had trouble deciding which rules to follow.

It was previously described that people in the USA who were at greater risk to be infected by the coronavirus were from the low socioeconomic portion of the society and were also African American or Hispanic. 22 23 24 25 It is important to take into account that this population was unable to discontinue the commute to their jobs, and they might have used a crowded public transportation, which did not allow physical distancing.

One quarter of the women were users of an IUD/IUS at the moment of their visit, and they came to the clinic for removal of their current device and insertion of a new one upon reaching the approved label time. Although we were aware about the recommendation of off-label extended use of the IUD/IUS made by different organizations, we decided to remove the one already in use and insert a new IUD because those women were already at the clinic. 26 27 28

We consider access to IUDs important at any time as they are highly effective contraceptives; however, access to it acquired greater importance during the pandemic. Keeping contraceptive clinics open and providing SRH services is important for the population even with a limited number of consultations, with reduced number of healthcare providers, and with fewer weekly working days. 1 12 26

Our study presents strengths and limitations. The main strength is the large sample size of women who voluntarily visited the clinic requiring an IUD. The main limitations are all respondents came from one center, one country, and most of them belonged to the middle socioeconomic class. This does not allow us to generalize our results; however, this is a group of women that received attention regularly from the Brazilian public sector.

It is important to reinforce the messages about the pandemic, especially among the underprivileged portion of the society. In addition, the COVID-19 pandemic offered new opportunities in the provision of LARC methods, focusing specifically on the needs of women with low socioeconomic status. Despite previous evidence showing that the provision of contraceptives is safe during the COVID-19 pandemic, there are many barriers worldwide. Some policy makers take advantage of the pandemic to impose restrictions and unnecessary regulations, such as those in some countries and some USA states. We consider our practice to continue offering IUDs during the pandemic is an important service to reduce inequality and improve the safety of women trough the pandemic. 1 9 29 30

## Conclusion

In conclusion, women without partners and with over 12 years of schooling had more information about COVID-19 and its impact on pregnancy. Women with high socioeconomic status and those with partners had a higher chance to respect physical distancing. We can also conclude that it is possible to provide IUDs/IUSs during the COVID-19 pandemic.
